# Effect of Soccer Boot Outsole Configuration on Translational Traction Across Both Natural and Artificial Playing Surfaces

**DOI:** 10.1177/23259671241259823

**Published:** 2024-08-08

**Authors:** Danyon Loud, Paul Grimshaw, Richard Kelso, William S.P. Robertson

**Affiliations:** † School of Electrical and Mechanical Engineering, The University of Adelaide, Adelaide, Australia; ‡ College of Health and Life Sciences, Hamad Bin Khalifa University, Doha, Qatar; Investigation performed at The University of Adelaide, Adelaide, Australia

**Keywords:** traction, lower limb, soccer, knee, cleats, outsole

## Abstract

**Background:**

Soccer boots are produced with different stud patterns and configurations to provide players with extra traction on specific surface types to minimize slipping and improve player performance. Excessive traction, however, can lead to foot fixation injuries, particularly anterior cruciate ligament tears.

**Purpose/Hypothesis:**

The purpose of this study was to explore the translational traction properties of 5 different outsole configurations moving in 4 different directions across both natural grass and artificial grass (AG) playing surfaces. It was hypothesized that longer studs or studs with an asymmetric shape would yield a higher traction coefficient compared with the recommended stud configuration for the given playing surface.

**Study Design:**

Descriptive laboratory study.

**Methods:**

A custom-built testing apparatus recorded the translational traction of 5 different soccer boots moving in an anterior, posterior, medial, or lateral direction on both natural grass and AG playing surfaces. A 3-way analysis of variance was performed to determine the effect of outsole configuration on the traction, and a post hoc Tukey analysis was performed to compare different outsole configurations with a control.

**Results:**

For the natural grass playing surface, the longer and asymmetric studs yielded a significantly higher (*P* < .05) traction coefficient on 75% of loading scenarios, while on AG, they yielded a significantly higher traction on 50% of loading scenarios.

**Conclusion:**

Some soccer boots yielded higher traction values compared with the recommended configuration.

**Clinical Relevance:**

The results highlight the importance of boot selection on different playing surfaces. Higher traction values could increase the injury risk for players due to excessive traction and foot fixation.

Soccer is one of the most popular sports played worldwide.^8^ Throughout a game of soccer, players perform kicking, acceleration, and deceleration movements and sharp changes of direction.^27^ Soccer boots are worn by players to aid in performing such maneuvers, as the studs, traditionally screwed into the outsole, add support and increase the contact area between the player and playing surface. In wetter conditions, players wear soft-ground (SG) boots with longer studs on the outsole. The longer studs are designed for deep penetration into the grass, allowing the soccer boot to “lock” itself into the root zone of the grass, which is less likely to become unstable, thus increasing traction.^30^ Improved manufacturing methods transitioned from the screw-in studs to molded plastic studs onto soccer boot outsoles. These molded studs were often shorter than SG studs and higher in number on boot outsoles. The basis of this design formed the firm-ground circular-shaped (FGC) boots that are prevalent today. In the late 1990s and early 2000s, firm-ground blade-shaped (FGB) studs were introduced, as manufacturers claimed they increased performance. While often the same height as FGC studs, the bladed design was utilized to increase the contact area between the boot and surface for linear movements and thus increase traction in the linear direction.^25^ The rise in popularity of artificial grass (AG) saw the evolution of outsoles to accommodate a larger number of smaller studs placed across the outsole, forming an AG design.^17^

To ensure that players do not slip on their playing surface, moderate levels of traction are often considered beneficial for player performance, allowing players to generate more power during acceleration movements, perform sharper turns, and decelerate more quickly.^33^ Translational traction is often defined as the ratio between the force along the direction of movement and the vertical force.^36^ In the literature, this is often indicated by an anterior movement along the longitudinal axis of the foot. While moderate levels of translational traction are generally accepted to be beneficial for athlete performance, excessive and minimal levels of translational traction along this axis can lead to foot fixation and slipping, respectively, which could cause lower limb injury.^9^ This is particularly evident with anterior cruciate ligament (ACL) injuries.

The stud geometry, length, and positioning on the soccer boot all affect player performance and potential injury risk, with reports suggesting that an athlete's ability to “free” themselves from the surface during adverse movements could prove beneficial when minimizing the risk of injury.^13^ Early studies from Bowers and Martin^1^ showed differences in translational forces of American football boots based on their wear, with shorter studs exhibiting lower levels of traction, which they described as “clearly detrimental to player performance.” In their 2010 study, Wannop et al^39^ examined the translational traction of 106 different pairs of used boots on a natural grass surface. These boots were divided into either edge, stud, or fin boots. Edge boots were categorized as those that had circular studs around the periphery of the boot with minimal interference along the center, while stud boots had a large number of circular studs dispersed over the outsole. Fin boots were similar to stud boots but used irregularly shaped studs over the outsole. While Wannop et al^39^ found no difference in translational traction between the 3 broad outsole configurations, the variation in design, placement, and size of studs within each category needs to be further examined. Similarly, Kuhlman et al^12^ found no difference in translational traction of shoes worn by National Football League players on AG at low to normal loads; however, at higher loads, they found higher levels of translational traction with boots that adopted a blade-shaped stud on the heel as opposed to circular studs on the heel.^12^ While translational traction is often measured in the anterior direction, movements such as a plant-and-cut motion occur in a lateral direction. De Clercq et al^5^ examined the tractional properties during this plant-and-cut movement in a laboratory setting, testing players in different outsole configurations. During their tests, they found that turf (TF) boots, akin to hockey shoes, provided less traction than AG and firm-ground boot variants for both wet and dry AG.

While some studies examined the impact of different outsole configurations on the translational traction properties under posterior-anterior movement and others on medial-lateral movement, there is currently no literature that examines the combination of both using the same testing apparatus. Thus, the aim of this study was to examine the different translational traction properties of different outsole configurations on both natural grass and AG under lateral and longitudinal loads. It was hypothesized that boots with longer studs, or studs with these asymmetric and aggressive patterns (ie, FGB boots), would yield a higher traction coefficient (TC) compared with the configuration recommended for use on each playing surface.^13,25^

## Methods

### Testing Apparatus

**Figure 1.**
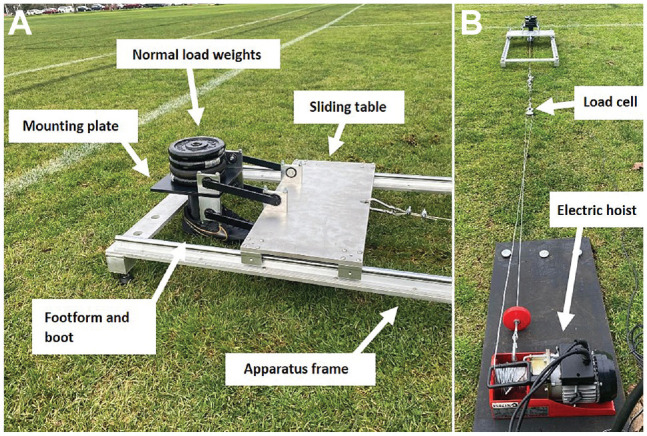
Photographs of the experimental setup on natural grass. (A) Footform and moving platform. (B) The connection of the hoist.

This study was conducted between November 2022 and February 2023. Ethics committee approval was not needed for this study as human participants were not involved. To test different outsole configurations on different surface types, a portable testing apparatus was developed to ensure that sports boots could be tested on actual playing surfaces and in multiple locations throughout these surfaces. The apparatus consisted of a footform beneath a mounting plate that was attached to a moving platform, constrained to linear motion by linear bearing rails mounted on an aluminum frame (Figure 1A). The mounting plate was connected to the moving platform using a 4-bar mechanism that ensured the footform remained level during each trial. An electric hoist (winch; model PA250D; Bada) pulled the moving platform along the test surface at a constant speed of 4 m/min (Figure 1B). This was the maximum operating speed of the winch in the high-torque setting. To record the tractional force, a 500-kg load cell (model STS Metric; SunScale; combined error of 0.025% of the output) was used to measure the tension in the hoist cable. The signal was passed through a data acquisition system (model USB-1616FS; Measurement Computing) that was recording at 150 Hz. The frame is leveled via 4 adjustable legs and then secured into place by 4 large pegs. Weights are added to the mounting plate to provide a total normal force of 148 N (inclusive of the weight of the plate) to the footform, which is mounted underneath. This normal force included 100 N of added weight, 45.5 N of the mounting plate, and 3.5 N from the footform and boots themselves.

**Figure 2.**
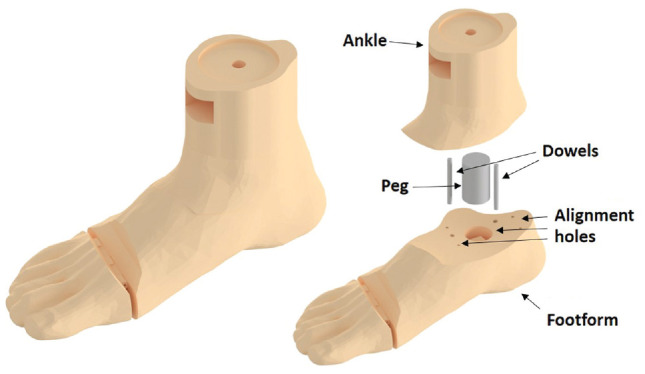
Assembled footform model used for testing (left) and unassembled footform model (right).

The footform, printed from acrylonitrile butadiene styrene filament, was adapted from a 3-dimensional scan of a male US size 9 foot. The footform, shown in Figure 2, was connected to a removable ankle joint via a series of pegs and dowels to ensure no movement of the foot relative to the ankle between each trial. The design of the separate foot and ankle allowed for easy interchange between boot types and loading direction.

### Playing Surfaces

**Figure 3.**
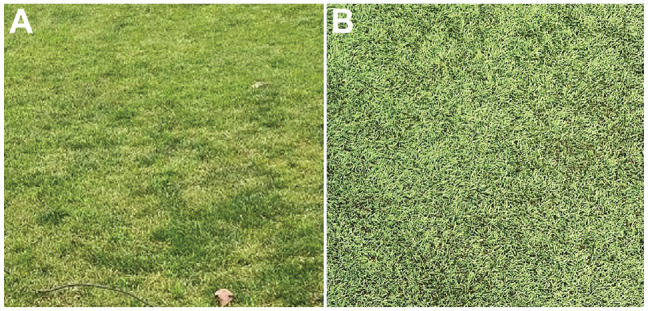
Testing surfaces used: (A) natural grass and (B) artificial grass.

Both natural grass and AG playing surfaces were utilized in this study. Both playing surfaces belonged to soccer clubs who compete in the top semiprofessional division in South Australia. The natural grass surface, shown in Figure 3A, consisted of a kikuyu base with ryegrass support. The surface underwent regular maintenance by ground staff, and its moisture content remained between 13% and 15% during the testing period. The AG surface, shown in Figure 3B, was a third-generation surface consisting of a 50-mm length Max S yarn, a styrene-ethylene-butylene-styrene performance infill, a quartz sand supporting infill, and finally a polypropylene ShockPad draining and shock absorption system.

A certain portion of each field was used for each group of 10 trials for each boot type and movement. Field locations were chosen to avoid areas of high use and wear (eg, the center circle and the penalty spot) as well as any sprinkler points or other interferences. Between each trial, debris was removed from the underside of the boot.

As the natural grass playing surface exhibits both temporal and spatial variations that can influence their mechanical properties, such as penetration and torsional resistance,^3^ 10 trials of each loading scenario were recorded to observe how the properties of the natural grass playing surface used in this study changed based on testing location.

### Boot Selection

**Figure 4.**
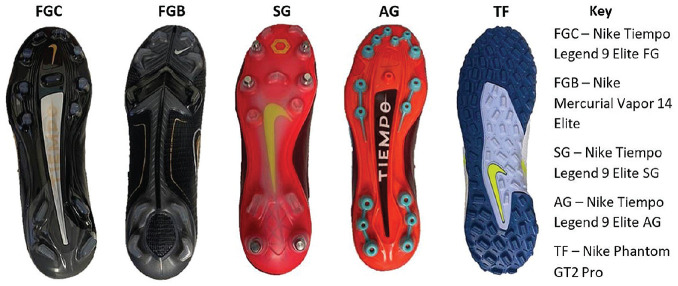
Outsole configurations of the different soccer boots used for testing. AG, artificial grass; FGB, firm-ground blade-shaped; FGC, firm-ground circular-shaped; SG, soft-ground; TF, turf.

There are a variety of soccer boots currently available on the market. These vary in outsole configuration, upper construction, material composition, and construction methods. While these properties differ considerably between boots, some brands offer the same type of boot with different outsole configurations (eg, SG, FGC, AG, and TF variants), with the upper construction, material, and shape of the boot remaining consistent throughout each variant. A 2020 study by the French media outlet Footpack showed that of 2520 professional players from the top 5 European leagues, Nike boots were worn by 1312 players, creating a 52.06% share of the market.^22^ Of the Nike soccer boot range, the Nike Tiempo model is the oldest boot still in production today, having first been released in 1984 with a leather upper and circular stud configuration. Because of its longevity and popularity, the latest iteration of the Nike Tiempo, the Nike Tiempo Legend 9 Elite boot (SG, FGC, and AG variants), was chosen for this study. The elite versions of these boots were selected to ensure premium materials and manufacturing quality between each boot. As the Nike Tiempo consists of a circular stud configuration, a different boot was chosen to represent the bladed outsole configuration (FGB). To ensure similar manufacturing methods, a Nike Mercurial Vapor 14 Elite boot was selected. Finally, a Nike Phantom GT2 Pro TF was selected as the TF option due to its availability. The boots used in this testing, all a men's US size 9, are shown in Figure 4.

### Testing Procedure

Before utilizing the testing apparatus in an outdoor setting, we calibrated both the STS 500-kg load cell and the apparatus as a whole. The STS load cell was calibrated with a series of known masses from 1 to 50 kg. The validity of the apparatus was confirmed by comparing dynamic friction coefficient values of common materials in a dry, unlubricated setting against the values obtained by Serway,^24^ as shown in Table 1. As the methodology was acceptable, different outsole configurations could be tested with various loading conditions.

**Table 1:** Dynamic Friction Coefficient of Materials Compared With Serway

Material Combination	Dynamic Friction Coefficient	
	With Testing Apparatus	From Serway^24^
Aluminum on steel	0.46	0.47
Steel on steel	0.55	0.57
Teflon on steel	0.05	0.04

**Figure 5.**
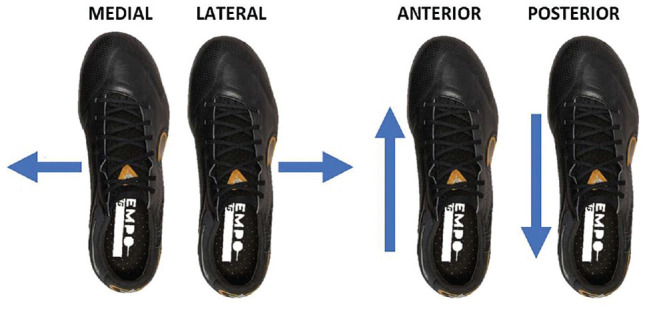
Different translational movements performed during the testing procedure.

Data such as air temperature, moisture content, humidity, and air pressure were recorded to ensure factors such as a change in moisture content could be analyzed if applicable. As mentioned, the trials within this study involved 5 different outsole configurations, across 2 surface types, performing 4 movements. These 4 movement types included translation of the right foot in the anterior, posterior, medial, and lateral directions, as shown in Figure 5.

Each trial was performed 10 times across the same patch of grass, with surface debris removed between each trial. Once trial 10 was concluded, the testing apparatus was moved to a new location on the playing surface, and a different outsole and movement type were chosen at random to minimize the risk of uneven wear of the boots over the testing period. The testing location each day was also chosen at random to ensure the boots did not deteriorate excessively on a single specific grass type. This testing procedure was repeated until all 5 outsole configurations, both surface types, and 4 movement directions were tested, resulting in a total of 400 trials. By performing repeated trials on the same part of the playing surface, the variation in traction observed across the playing surface was minimized, according to Loud et al.^16^

### Data Processing

The voltage recorded from the data acquisition system was then imported to a custom MATLAB (MathWorks) script that filtered the data using a moving average filter over 3 data points and converted it to force data based on calibration. The moving average was used to smooth data from the step response given by the load cell while maintaining the overall trend. The smoothing produced an amplitude attenuation of <1% of the maximum recorded force.

Zanetti et al^41^ suggested that, for an analysis of the likelihood of injury, the peak force should be examined for each trial. To ensure a dimensionless approach, the TC was compared instead of the peak force. The TC is defined by Wannop et al^35^ as the ratio of the horizontal force (traction force) relative to the normal force. Two key variations of this TC were recorded for analysis. The first was the maximum TC experienced in trial 1, as this is often the peak level of traction whereby a noncontact lower limb injury is most likely to occur.^41^

### Statistical Analysis

The mean maximum TCs for trials 2 to 10 were used for statistical analysis between different loading conditions. These values were first grouped based on surface type, movement, and outsole configuration before being compared using a 3-way analysis of variance. Data were examined for distribution using a Shapiro-Wilk test, and some data were found to be approximately nonnormal in terms of distribution. To observe the effects of the initial and subsequent changes in trials, Brown and Forsythe^2^ suggested that when distributions are slightly nonnormal, the Levene median test for the homogeneity of variance should be used instead of the Levene mean test. Based on this test, as well as fulfilling all other assumptions, the 3-way analysis of variance was run to determine the impact of outsole configuration, surface type, and movement type, as shown in Table 2.

**Table 2:** Influence of Outsole Configuration, Surface Type, Movement Type, and Their Combinations on the Tractional Coefficient for Trials 2 to 10

Factor	*P*	Partial η^2^
Outsole configuration	<.001	0.644
Surface type	<.001	0.751
Movement type	<.001	0.172
Outsole configuration × surface type	.039	0.041
Outsole configuration × movement type	<.001	0.243
Surface type × movement type	<.001	0.126

The significance level in Table 2 demonstrated that all 3 testing factors, as well as their combinations, were statistically significant and had a key impact on the amount of tractional force being produced. From the partial η^2^ values in Table 2, it is seen that the surface type, followed by the outsole configuration, had the greatest impact on the TC, and thus they formed the basis for analysis.

All statistical analyses were performed in SPSS Statistics (Version 27; IBM). The threshold for statistical significance was set at *P* < .05.

## Results

### Influence of Natural Grass Surface Wear on TC

**Figure 6.**
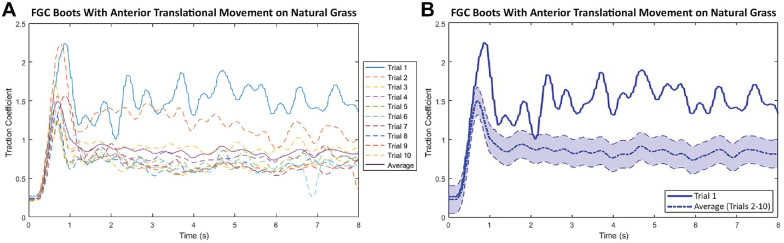
(A) Unfiltered traction coefficient for each of the 10 trials and (B) traction coefficient for trial 1 and the mean traction coefficient for trials 2 to 10 (shaded band indicates standard deviation) for firm-ground circular-shaped (FGC) boots moving in the anterior direction on natural grass.

The alterations in TC through 10 trials for FGC boots on the natural grass playing surface are demonstrated in Figure 6.

The TCs for the initial trials were larger throughout the duration of loading, particularly around their maxima, than they were for the remaining trials. The remaining trials (trials 2-10), however, showed small variation among the values for the TC, highlighted by the small standard deviation band in Figure 6B.

### TC of Different Outsole Configurations on Multiple Playing Surfaces

**Figure 7.**
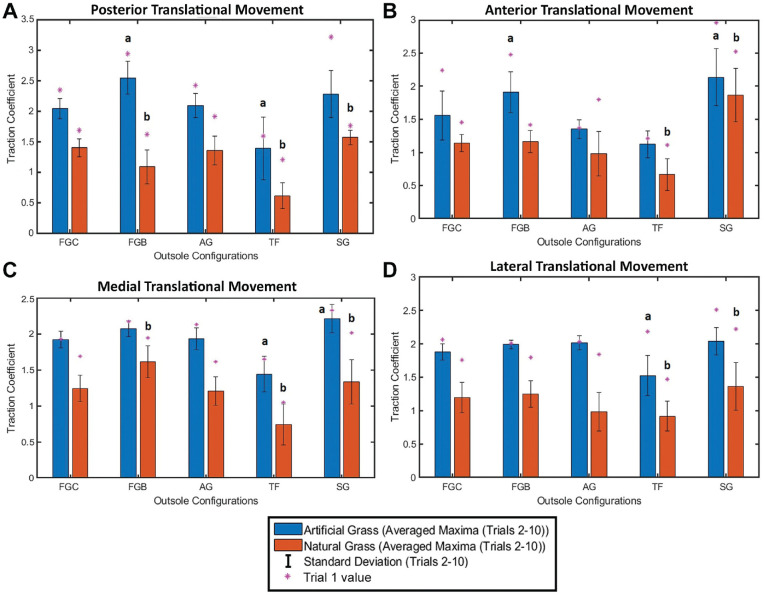
Effect of outsole configuration and surface type on the traction coefficient for boots moving in the (A) posterior, (B) anterior, (C) medial, and (D) lateral directions on the different playing surfaces. ^a^Significant difference compared with artificial grass (AG) boots (*P* < .05). ^b^Significant difference compared with firm-ground circular-shaped (FGC) boots (*P* < .05). FGB, firm-ground blade-shaped; SG, soft-ground; TF, turf.

The summary statistics of the mean maximum TCs for trials 2 through 10 and the maximum TC for trial 1 for each movement type on both surfaces are detailed in Figure 7. Using these graphs, we can observe the impact of outsole configuration as well as surface type. A post hoc Tukey test was used to compare the stud configurations against a standardized control boot for a particular surface. For the natural grass surface, the FGC boot was selected for comparison because of its popularity, while the AG boot was used on AG because it was specifically designed for that playing surface. The statistically significant results are highlighted in Figure 7, with full statistical results for the Tukey tests included in Appendix Tables A1 and A2. For all movement types, the TF configuration produced significantly less traction than the control boot for one or both of the playing surfaces. Conversely, the SG configuration produced larger levels of traction for ≥1 surfaces for all movement types. These 2 configurations indicate the maximum and minimum stud lengths used for testing, highlighting the importance of length for level of traction.

## Discussion

The results from this investigation highlight the importance of playing surface and movement direction of the level of traction obtained for each outsole configuration. As demonstrated in Figure 7, configurations with generally longer or irregularly shaped studs generated more traction than elliptical and shorter stud patterns. Because of the composition of the playing surface, the amount of traction generated by each configuration was higher on the AG playing surface compared with the natural grass surface.

### Influence of Surface Wear on Traction

Studies performed by Caple et al^3^ and Wannop et al^37^ showed that the TC varies on the same natural grass playing surface. This may be caused by inconsistencies in root zone growth, irregular maintenance, pests, and diseases that impact the species of grass or other outside factors. As shown in Figure 6A, the initial trials often exhibit a higher tractional resistance than subsequent trials. This higher value experienced in the initial trial can be explained by the initial penetration of an intact root zone system. This is more prevalent on the natural grass playing surface consisting of kikuyu and ryegrass, both of which generally have large stolon coverage and length, thus providing more resistance to penetrative movement.^6^ The initial test, however, disrupts the root zone and tears these stolons, reducing the resistance in the natural grass surface in subsequent tests. In a similar way on the AG surface, the initial trial displaces the rubber infill pellets from the boot's path, providing less resistance for subsequent trials.

These inconsistencies in surface traction were also reported on natural and artificial American football fields by Wannop et al.^37^ In the current study, the outlier behavior of trial 1, shown in Figure 6B, was seen consistently across all movements tested. Statistical analysis was performed on the mean data across trials 2 to 10, justified by the lower variance exhibited when trial 1 was removed. The mean of the maximum TCs (trials 2-10) was used to compare the traction performance between configurations. This approach was supported by the study of Loud et al,^16^ who found that there was a larger coefficient of variation of the TC over the entire trial period for tests that were performed on different locations of the same playing surface when compared with trials performed on the same location of the playing surface. Contextually, this also represents some level of wear on the playing surface, as is the case with a majority of soccer pitches worldwide.

### Effect of Outsole Configurations

As evidenced in Figure 7, the type of boots a player chooses to wear has an impact on the tractional loads they experience on both types of playing surfaces and over a variety of translational movements. These results are consistent with others reported in the literature.^19,25,28^ These authors, coupled with Cooper^4^ and Thomson et al,^30^ explored the impact of outsole configuration on translational traction; however, their loading mechanisms were limited. Both Stefanyshyn et al^26^ and Thomson et al^30^ applied an anterior movement to a footform with a level of plantarflexion. While this particular movement is common when performing straight-line running, it is far more common for players to injure themselves when slowing down or changing direction, where the foot is in a more neutral position or with some level of dorsiflexion.^12^ The irregular shape of boots, coupled with the nonsymmetric outsole configuration, results in a translational traction that differs with respect to the direction of loading. This asymmetry, and the effect of movement direction, is not widely researched within the same study among the literature.^21^

Posterior translational movement of the foot (Figure 7A) indicates a potential sudden takeoff for a player, a loading scenario not commonly linked to ACL injury, but rather linked to performance, particularly of forwards and defenders. The low tractional properties of TF boots, on both natural grass and AG, indicate the potential for players to slip during this takeoff. Conversely, a higher TC value of the FGB boots on AG could indicate potential for a more explosive takeoff, as players are able to generate more momentum because of the higher traction.^19^ Similar results were obtained by Shorten et al^25^ whereby a blade-shaped stud pattern achieved higher values of translational traction during posterior movements. On natural grass, however, FGB boots produced a lower TC compared with FGC boots, which could be caused by to the orientation of the rear chevron studs. As these studs are aligned such that the contact area when moving posteriorly is small, the FGB boots are better equipped to cut through the root zone of the natural grass, minimizing the resistance and lowering the TC.

Foot fixation during a sudden deceleration or change of direction is highlighted by Ireland^10^ and Murray and Fleming^20^ as a potential mechanism for ACL injury. The tibia becomes fixated with respect to a moving femur, which can then lead to excessive valgus or hyperextension of the knee, placing the ACL under strain. Anterior traction of boots is particularly important in the moderation of foot fixation of soccer players. As shown in Figure 7B, SG boots had a higher TC value compared with both controls on natural grass and AG, indicating a greater likelihood of foot fixation and potential injury. Similarly, on AG, the bladed stud configuration experienced a higher TC value compared with the firm-ground equivalent and the AG control, indicating an increased potential injury risk. It can be noted in Figure 4, however, that the FGC and FGB boots both adopt noncircular rear foot studs of differing heights, with FGC studs sitting at 12 mm in length, while the FGB studs are 15 mm in length. The impact of stud height can also be observed in the similarities of SG configurations (15 mm in height) and FGB results on AG and the low TC of the TF boots, whose studs only measure 5 mm in height. This increase in traction with increasing stud height was an observation shared by Müller et al^19^ and Twomey et al.^32^

While not as common as the standard sidestep cut maneuver, the crossover cut occurs when the athlete's direction of travel is toward the same side of the body as the pivoting leg.^15^ This crossover cut is represented by the medial translation of the foot, and while not likened to a mechanism for ACL injury, it does provide certain performance benefits as players often utilize a sharp change of direction to elude opponents. Figure 7C shows that TF boots produced a lower TC value on both playing surfaces, indicating an increased likelihood for slipping when performing a crossover cut maneuver. It also shows the impact of stud length, as SG boots yielded a higher TC on both surface types. The increased contact area of the chevron studs in the FGB configuration led to a higher TC on natural grass compared with the FGC boots, indicating significantly better grip on the surface. Given the uncommon nature of a crossover cut maneuver, investigations of such tractional properties among literature are limited. Cooper^4^ examined the translational traction of National Football League cleats moving in the medial direction along FieldTurf AG of a similar composition to the TF shown in Figure 3B. While the boots tested were designed for a different sport, the number and generic shape of the studs were similar to those used in this procedure. The TC values shown in Figure 7C were similar in magnitude to those found by Cooper^4^ with boots of a comparable design, validating the testing procedure of the medial movement.

The lateral movement of the footform was used to simulate a sidestep cut motion, as this is a movement commonly associated with noncontact ACL injury. Ireland^10^ suggested that when a player performs a sidestep cut movement, there is a potential for their foot to become fixed to the playing surface. This can cause external tibial rotation and internal femoral rotation as the player propels from the movement, creating a collapse of the knee and placing the ACL under stress from excessive knee valgus. As demonstrated in Figure 7D, there was no outsole configuration that provided significantly more lateral traction compared with the others. TF boots, however, exhibited low levels of traction on both tested playing surfaces, indicating an increase slipping potential. While the SG configuration produced the highest TC value on both surfaces, only the result on natural grass was significant. In similar observations, Sterzing et al^28^ and Müller et al^18^ found that SG boots induced a higher level of lateral traction compared with FGB boots during lateral movement; however, their testing methods differed from those of the present study in terms of movement speed, duration, and boot selection.

### Effect of Surface Type

As indicated in Table 1, the playing surface had the largest impact on the translational traction, with AG surfaces providing higher traction levels in every movement. This increase in traction is similar to the increase in rotational traction observed by Rouch et al^23^ during running and cutting movements. In a more recent study, Thomson et al^29^ found that, for 55.5% of outsole configurations tested, there were significant differences between the translational traction on artificial and natural grass playing surfaces. The context behind this increase in traction is well explained by Kent et al,^11^ who suggested that when a translational force is applied to a boot, the boot can undergo 1 of 3 scenarios: it can hold its position relative to the surface, it can tear through the playing surface, or it can slide through the playing surface. Kent et al^11^ then suggested that a key characteristic of artificial playing surfaces is that they do not, under normal loading, tear. Thus, AG lacks a potential mechanism for injury mitigation often seen on natural TF. This phenomenon is shown in Figure 7C, whereby trials 1 and 2 tore through the root zone of the natural grass, lowering the tractional force needed for the remaining trials and thus minimizing the potential for foot fixation and an increased risk of injury. This effect can be observed in Figure 7, whereby for 100% of the natural grass data, the initial trial yielded a higher TC than the mean value plus 1 SD. For the AG data, 45% of the trials yielded an initial TC less than the mean value plus 1 SD.

### Limitations and Considerations

While the present study was able to observe the impact of outsole configuration and surface type on the TC for a variety of movement types, there were some limitations present. To ensure consistency among trials, and to ensure the entire outsole configuration was in contact with the surface, each movement was performed with a neutral foot angle. This was the same approach used by Grund and Senner,^9^ Villwock et al,^34^ and Kuhlman et al^12^ to ensure engagement of all studs on the sole. It is, however, highly unlikely that the loading surface of the outsole is uniform across a particular movement; thus, the impact of ankle angle during loading must be taken into consideration.

The present research was also limited in terms of the normal load applied to the apparatus and the translational speed of the boot. A normal load of 146 N was applied to the footform, a load significantly lower than those in previous studies^9,29,31^; however, the researchers were able to achieve full stud penetration for all configurations on both the natural grass and AG playing surfaces under this load. The impact of normal load on the TC has been discussed within the literature, with Wannop and Stefanyshyn^38^ stating that there is a linear relationship between TC and normal load, finding that the order of outsole configuration that provided the most and least traction did not change for each normal load. These results were supported by the works of Torg et al,^31^ Bowers and Martin,^1^ Warren,^40^ and Livesay et al.^14^ This demonstrates that as long as the normal load applied to the testing procedure is consistent, the relationships between each testing variable will remain the same. Because there was adequate stud penetration, and the normal load was applied in a consistent manner, the methodology was considered appropriate for comparison between stud configurations and associated movements. While the translational speed of 4 m/min is below the typical speeds that players experience in a match, the speeds were approximately equal to those used by Cooper.^4^ A higher translational speed may require the use of a dynamic measure of normal force, which would complicate the mechanism design and data acquisition.

There are a number of variations within AG playing surfaces, from sand-based, short-pile grasses used in hockey to the longer, third-generation AG playing surfaces that are utilized by sports such as soccer or American football. Currently, the international governing body for soccer (Fédération Internationale de Football Association) will approve play on only the third-generation artificial TF that meets specific requirements regarding usage and maintenance.^7^ Although there are a number of different AG playing surface styles, there is only one surface type that is approved for use with soccer. Therefore, the results obtained for this investigation were classified as AG results.

## Conclusion

Translational traction has a key impact on the health, safety, and performance of soccer players. The results of this study showed that the outsole configuration, playing surface, and loading condition can all have an impact on the tractional loads experienced by players. Boots with longer or irregularly shaped stud patterns often resulted in larger levels of traction in anterior, posterior, and medial movements across natural grass and AG types. This increase in traction could lead to an increased risk of foot fixation and potential lower limb injury. This study highlighted the tractional properties of movements that are not widely reported in the literature, such as the posterior movement involved in an acceleration, which could lead to potential slipping, or the medial translation of the foot in a cross-cut maneuver, whereby excessive traction could place the knee under extreme load, causing potential injury.

## Appendix

**Table A1:** Post Hoc Tukey Analysis of the Traction Coefficient of Different Outsole Configurations for Different Movements on Natural Grass^*a*^

Movement and Outsole Type	Mean Difference (vs FGC)^*b*^	*P*
Anterior		
AG	0.130	.729
FGB	−0.055	.985
SG	−0.779	**<.001**
TF	0.443	**.001**
Posterior		
AG	0.012	≥.999
FGB	0.278	**.004**
SG	−0.200	.066
TF	0.754	**<.001**
Medial		
AG	−0.013	≥.999
FGB	−0.421	**<.001**
SG	−0.143	.466
TF	0.495	**<.001**
Lateral		
AG	0.156	.222
FGB	−0.112	.547
SG	−0.225	**.028**
TF	0.219	**.035**

a Boldface *P* values indicate a statistically significant difference vs FGC (*P* < .05). AG, artificial grass; FGB, firm-ground blade-shaped; FGC, firm-ground circular-shaped; SG, soft-ground; TF, turf.

b The FGC configuration was used as the control for the comparisons on natural grass.

**Table A2:** Post Hoc Tukey Analysis of the Traction Coefficient of Different Outsole Configurations for Different Movements on Artificial Grass^*a*^

Movement and Outsole Type	Mean Difference (vs AG)^*b*^	*P*
Anterior		
AG	−0.557	**<.001**
FGB	−0.206	.502
SG	−0.786	**<.001**
TF	0.226	.411
Posterior		
AG	−0.443	**.026**
FGB	0.049	.997
SG	−0.188	.696
TF	0.701	**<.001**
Medial		
AG	−0.141	.453
FGB	0.009	≥.999
SG	−0.282	**.014**
TF	0.494	**<.001**
Lateral		
AG	0.025	.996
FGB	0.135	.299
SG	−0.027	.995
TF	0.487	**<.001**

a Boldface *P* values indicate a statistically significant difference vs AG (*P* < .05). AG, artificial grass; FGB, firm-ground blade-shaped; SG, soft-ground; TF, turf.

b The AG configuration was used as the control for the comparisons on artificial grass.
